# Lipoxygenase Inhibition by Plant Extracts

**DOI:** 10.3390/biom11020152

**Published:** 2021-01-25

**Authors:** Melita Lončarić, Ivica Strelec, Tihomir Moslavac, Drago Šubarić, Valentina Pavić, Maja Molnar

**Affiliations:** 1 Faculty of Food Technology Osijek, Josip Juraj Strossmayer University of Osijek, Franje Kuhaca 18, 31000 Osijek, Croatia; melita.loncaric@ptfos.hr (M.L.); ivica.strelec@ptfos.hr (I.S.); tihomir.moslavac@ptfos.hr (T.M.); drago.subaric@ptfos.hr (D.Š.); 2 Department of Biology, Josip Juraj Strossmayer University of Osijek, Ulica cara Hadrijana 8/A, 31000 Osijek, Croatia; vpavic@biologija.unios.hr

**Keywords:** lipoxygenase, plant extracts, polyphenols

## Abstract

Lipoxygenases are widespread enzymes that catalyze oxidation of polyunsaturated fatty acids (linoleic, linolenic, and arachidonic acid) to produce hydroperoxides. Lipoxygenase reactions can be desirable, but also lipoxygenases can react in undesirable ways. Most of the products of lipoxygenase reactions are aromatic compounds that can affect food properties, especially during long-term storage. Lipoxygenase action on unsaturated fatty acids could result in off-flavor/off-odor development, causing food spoilage. In addition, lipoxygenases are present in the human body and play an important role in stimulation of inflammatory reactions. Inflammation is linked to many diseases, such as cancer, stroke, and cardiovascular and neurodegenerative diseases. This review summarized recent research on plant families and species that can inhibit lipoxygenase activity.

## 1. Introduction

Lipoxygenase (LOX) was discovered as an enzyme that catalyzes oxidation of carotene destruction, as first reported by Bohn and Haas. The enzyme was named carotene oxidase. Throughout the years, LOX has been identified in a variety of ways. From 1932 to 1940, names such as unsaturated lipid oxidase, lipoxidase, fat oxidase, and lipid oxidase were proposed [1]. In 1940, it was recognized as the same enzyme. All previously used names were consolidated and the enzyme was officially named lipoxygenase [2].

Lipoxygenases (LOXs) are a family of monomeric proteins that catalyze oxidation of polyunsaturated fatty acids (PUFA) (linoleic, linolenic, and arachidonic acid) to produce hydroperoxides [3]. LOXs are widely spread in animal, plant, and fungi kingdoms, as well as in cyanobacteria [4]. Moreover, 5-LOX is ubiquitous in mammalians and oxygenates carbon-5 on arachidonic acid, while 9-LOX and 13-LOX are plant LOXs that catalyze oxygenation of linoleic and linolenic acid [4,5].

Lipoxygenases are present in the human body and play an important role in the stimulation of inflammatory reactions. Excessive amounts of reactive oxygen species can cause inflammation that stimulates a release of cytokines and subsequent activation of LOXs. Inflammation is linked to many diseases, such as cancer, stroke, and cardiovascular and neurodegenerative diseases. LOXs are involved in the synthesis of prostaglandins and leukotrienes. They are associated with disease development and their inhibition is considered as a crucial step in disease prevention [6].

LOXs can be abundantly found in cereals (wheat, corn, oat, rye, barley), legumes (mung beans, green beans, peas, navy beans, soybeans), and potato tubers [7,8]. Products generated by LOX pathways have various functions. LOX is used as storage protein during vegetative growth. In addition, LOX is related to the mobilization of storage lipids during germination [9]. LOX also plays an important role in the production of protective components (jasmonates, divinyl ethers, leaf aldehydes), which helps protect plants from insects and pathogens or during abiotic stress [10,11]. On the other hand, LOX reactions with unsaturated fatty acids can produce off-flavors/off-odors and cause food spoilage. Due to the undesired components of the LOX pathway, research was carried regarding lipoxygenase inhibition [3,12].

Plant phytochemicals play an important defensive role, which could be beneficial in the prevention of diseases caused by oxidative stress. Various plants have been used for thousands of years as medicine. Nowadays, many drugs are produced via isolation from natural sources and there is increasing interest in plant derived therapeutics [13].

The aim of this review was to summarize the plant extraction methods for the best inhibitory activity of lipoxygenase enzymes recently published.

## 2. Lipoxygenase Inhibition by Plant Extracts of Family Apiaceae

Apiaceae (*Umbelliferae*) is an angiosperm family that consists of 454 genera and roughly 3700 species. Family members are used as food, spices, as well as medicinal plants, since they are rich in polyphenols. They possess aromatic nature and wide range of chemical compounds, but some of them can also be toxic [14]. Many compounds of the Apiaceae family possess biological activities, such as antibacterial, vasorelaxant, hepatoprotective, cyclooxygenase inhibitory, antitumor activities, and many others [15]. A short overview of the extraction on different plant parts of the Apiaceae family, with the investigation of their inhibitory activity against LOX, is shown in Table 1.

Muleya et al. isolated five diterpenoids from the roots of the plant *Alepidea amatymbica* [16]. Inhibition of 15-soybean lipoxygenase were performed and 14-acetoxo-12-oxokaur-16-en-19-oic acid showed high inhibitory activity (EC_50_ = 19.10 µg/mL), slightly lower than the control compound indomethacin (EC_50_ = 17.22 µg/mL). Four other compounds showed poor-to-moderate LOX inhibition in EC_50_ range 25.98–81.18 µg/mL.

Inhibition of 15-lipoxygenase with seed extracts of *Apium graveolens* and *Petroselinum crispum* was performed by Danciu et al. [17]. It was found that 86.15 mg of *Apium graveolens* and 69.45 mg of *Petroselinum crispum* extract is necessary to inhibit 50% of 15-lipoxygenase activity. Phytochemical compositions of obtained methanolic extracts were estimated and nine components were detected. Apigenin and apigenin glucoside were the most prevalent compounds in both extracts. Aerial parts of *Bupleurum marginatum* were extracted in two different solvents, methanol and dichloromethane [18]. Both extracts showed 5-lipoxygenase inhibitory activity at concentration of 25 µg/mL. Methanolic extract showed higher IC_50_ value (45.28 µg/mL) than the extract obtained in dichloromethane (25.92 µg/mL). Phytochemical studies showed that *B. marginatum* extracts are rich in lignans, flavonoids, saikosaponins, and other terpenoids. Methanol extract possessed more flavonoids, while dichloromethane extract had more lignans and terpenoids. The potential ability of soybean lipoxygenase inhibition was also tested by the methanolic extract of *Scandix pecten-veneris* [19]. Enzyme inhibition was measured on five different concentrations of plant extracts, of 100, 200, 300, 600, and 900 µg/mL, and obtained results showed 3.50, 16.42, 34.62, 44.56, and 77.49% of LOX inhibition, respectively. Total phenolic content of examined extract was 377.94 mg equivalent (GAE)/g extract. It was reported that coumarin umbelliprenin found in the plant family Apiaceae strongly inhibits lipoxygenase [24]. Water extract of *Pituranthos chloranthus* showed strong lipoxygenase inhibition with IC_50_ value of 0.02 mg/mL [20]. Total polyphenol content of the extract was 1.36 mg GAE/g. Paun et al. extracted aerial parts of *Eryngium planum* in 50% ethanol with water (*v*/*v*) [21]. The obtained extract was subjected to lipoxygenase inhibition testing and showed high inhibitory activity with an IC_50_ value of 31.30 µg/mL. The polyphenol profile of *E. planum* showed a high concentration of flavonoids, especially isoquercetin (36.11 µg/mL) and rutin (290.52 µg/mL).

To increase polyphenol production, Złotek and co-workers used jasmonic acid (1, 10, 100 µM) and yeast extract (0.01, 0.1, 1%) as elicitors [22]. The best results were observed in *Levisticum officinale* leaves treated with 10 µM jasmonic acid, where total polyphenol content increased by 55% in comparison with the control sample. All extract showed the ability to inhibit lipoxygenase, but inhibition rate of elicited leaves did not differ significantly from the control. Similar research was conducted by Majdoub et al., who monitored the effect on 2 mM Zn on polyphenol amount in *Anethum graveolens* (dill)***,*** *Pimpinella anisum* (anise), and *Foeniculum vulgare* (fennel) [23]. Total polyphenol content increased in dill and anise plants, while in fennel, a decrease of phenol content was observed. Treated and non-treated samples had the ability to inhibit 5-lipoxygenase. Anise showed strong lipoxygenase inhibitory activity, with IC_50_ of 0.035 and 0.015 mg/mL for Zn non-treated and treated plants, respectively. In the case of dill, no significant difference was observed in LOX inhibition before (IC_50_ = 0.043 mg/mL) and after (IC_50_ = 0.041 mg/mL) treatment with Zn. Fennel showed higher IC_50_ value in the sample treated (IC_50_ = 0.062 mg/mL) with Zn, compared to non-treated (IC_50_ = 0.052 mg/mL) one.

Among investigated extracts of the Apiaceae family, the best inhibitory activity of lipoxygenase was noticed in water extract of *P. chloranthus*. Further, strong inhibitory activity (IC_50_ = 25.92 µg/mL) of *B. marginatum* extract could be related to phytochemicals identified in dichloromethane extract: isorhamnetin and its glycoside narcissin, quercetin and its glycoside rutin and isoquercitrin, (3,4-dimethoxybenzyl)-2-(3,4-methylenedioxybenzyl) butyrolactone and marginatoxin [25].

## 3. Lipoxygenase Inhibition by Plant Extracts of Family Asteraceae

Asteraceae is the biggest family of flowering plants and counts about 1100 genera and 20,000 species. Many plants from the Asteraceae family are used as traditional medicine due to the possession of numerous phytochemical components, such as polyphenols and diterpenoids. Antifungal, antibacterial, insecticide, anti-inflammatory, and antitumor activities were investigated in the Asteraceae species [26]. Many plants from this family were assayed on the inhibitory activities against the lipoxygenase enzyme (Table 2).

Bhat et al. extracted leaves of *Artemisia nilagirica* in three different solvents (hexane, methanol, and ethanol) [27]. All extracted inhibited lipoxygenase, but the most effective was the methanolic extract, with an IC_50_ value of 128 µg/mL, followed by hexane extract (196 µg/mL). Ethanolic extract showed poor inhibition of 21%. Burlec et al. extracted two plants from the Asteraceae family (*Chrysanthemum indicum* and *Tagetes erecta*), in four different organic solvents (chloroform, dichloromethane, hexane, and methanol) in order to investigate extract inhibition ability of 15-lipoxygenase [28]. The strongest enzyme inhibition of *Chrysanthemum indicum* was in chloroform extract with EC_50_ value of 26.06 µg/mL while *Tagetes erecta* methanol extract showed similar inhibition (EC_50_ = 25.85 µg/mL). The highest phenolic concentration was obtained in *T. erecta* chloroform extract, while dichloromethane extract of *C. indicum* showed the highest polyphenol content. Five different organic solvents (methanol, dichloromethane, ethyl acetate, *n*-hexane, and *n*-butanol) were employed in the extraction of *Vernonia oligocephala* roots [29]. All extracts were investigated for their lipoxygenase inhibition potential. Among all extracts, only butanol extract showed considerable LOX inhibition (IC_50_ = 132.20 µg/mL). Phytochemical composition analysis of the roots showed the presence of saponins, terpenoids, flavonoids, glycosides, phenolic compounds, and steroids. Methanol extract had the highest flavonoid (97.35 mg quercetin equivalent (QE)/g of extract) and phenolic (113.11 mg GAE/g of extract) content. Yildirim et al. extracted different parts of *Tanacetum cilicicum* (capitula, leaves, and stem) in different solvents (ethanol, *n*-hexane, chloroform, and ethyl acetate) [30]. Extract of capitula in ethyl acetate showed significant LOX inhibition, with an IC_50_ value of 9.44 µg/mL, which is even greater than the standard indomethacin (IC_50_ = 22.39 µg/mL). Ethyl acetate was also proven to be the best choice for the leaves extraction, in respect to LOX inhibition (IC_50_ = 85.20 µg/mL), while the best inhibition among stem extracts was observed for chloroform extracts (IC_50_ = 112.40 µg/mL). Total polyphenol content in extracts varied 6.10–175.60 mg GAE/g of dry weight, while total flavonoid content was in the range of 6.13 to 30.48 mg QE/g of dry weight. Phenolic contents were the highest in all ethyl acetate extracts. Water extract of *Artemisia vulgaris* investigated by Nasr et al. had a high ability of lipoxygenase inhibition (IC_50_ = 40.00 µg/mL) [20]. Total phenolic content of aqueous extract was 0.91 mg GAE/g of extract.

Ethanolic extract of *Cnicus benedictus* investigated by Paun et al. also showed very good ability of lipoxygenase inhibition (IC_50_ = 52.70 µg/mL) [21]. Total flavonoid content in extract was 0.47 mg QE/mL and total phenolic content was 2.65 mg GAE/mL. High concentrations of phenolic acids were determined (especially sinapic acid and chlorogenic acid). Further, methanolic extract of *Matricaria chamomilla* showed good inhibition of lipoxygenase with EC_50_ value of 166.32 mg/mL [17]. Total polyphenol content (458.10 µg GAE/mL of extract) and total flavonoids (342.40 µg/mL of extract) of extract were determined. Özek et al. investigated LOX inhibition ability of essential oils and methanolic extracts from *Galatella villosa* and *Galatella tatarica* [31]. The 5-LOX was inhibited with both extracts and essential oils. The oils proved to be better inhibitors with percentages of inhibition of 45.0 and 57.0% for *G. tatarica* and *G. villosa,* respectively. Both methanolic extracts showed poor 5-LOX inhibition of 29.0% and 19.0%, respectively. Content of total phenolic compounds did not differ significantly in the extracts. Similar research was conducted with the oil and extract of *Cota fulvida* and similar results were obtained [32]. The essential oil inhibited 5-LOX for 53.7% in comparison to poor inhibition of 23.9% of methanol extract.

The highest capacity of LOX inhibition was achieved with ethyl acetate extract of *T. cilicicum* capitula. Among all extracts, this one showed high phenolic content (174.10 mg GAE/g of extract) as well as flavonoid concentration (26.94 mg QE/g of extract).

## 4. Lipoxygenase Inhibition by Plant Extracts of Family Clusiaceae

The Clusiaceae family has approximately 400 species widespread in Africa, Asia, Polynesia, and South America. *Garcinia* is the most numerous genus of Clusiaceae, and its plant parts, such as fruit, leaves, stems, and flowers, are commonly used for the treatment of inflammation and oxidative stress. Some plants also possess biological activities, such as antioxidant, anti-inflammatory, and antibacterial [33]. Potential lipoxygenase inhibition was investigated for extract of *Garcinia* plants (Table 3).

Sari and Elya performed LOX inhibition assay on the stem bark extracts of *Garcinia porrecta* Laness [34]. Extraction was conducted in different organic solvents (methanol, *n*-hexane, and ethyl acetate). LOX inhibition activity of extracts with IC_50_ values were 0.23, 0.52, and 4.87 μg/mL for methanol, ethyl acetate, and *n*-hexane extracts, respectively. Total flavonoids of methanolic extract were equal to 5.66 mg QE/g. Extraction of *Garcinia hombroniana* Pierre leaves in *n*-hexane, methanol, and ethyl acetate was performed by Marlin and Elya [35]. It was proven that all leaf extracts are excellent LOX inhibitors with IC_50_ values of 0.13, 1.31, and 2.05 μg/mL for ethyl acetate, methanol, and *n*-hexane extracts, respectively. Determined flavonoid content of the most active ethyl acetate extract was 42.00 mg QE/g sample. LOX inhibition potential of *Garcinia lateriflora* Blume leaf extracts was investigated [36]. IC_50_ values of the LOX inhibition were 0.69, 0.79, and 1.32 μg/mL for methanol, ethyl acetate and *n*-hexane extracts, respectively. The most active extract was the methanolic one, with total flavonoid content of 6.29 mg QE/g of extract. Further, *Garcinia kydia* Roxburgh leaves were extracted with three previously mentioned solvents [37]. Obtained results on LOX inhibition had IC_50_ values of 0.21, 0.55, and 3.57 μg/mL ethyl acetate, methanol, and *n*-hexane extracts, respectively. Total flavonoid content of 30.65 mg QE/g of extract was determined in the most active ethyl acetate extract.

Four mentioned *Garcinia* plants from the Clusiaceae family were extracted in the same solvents, and all showed strong inhibition of the LOX enzyme. It could be noticed that the best results are obtained with methanol and ethyl acetate extracts, while *n*-hexane extracts showed a slightly lower inhibition activity. The strongest LOX inhibitor was the ethyl acetate extract of *G. hombroniana* with the highest amount of total flavonoids among all investigated extracts.

## 5. Lipoxygenase Inhibition by Plant Extracts of Family Fabaceae

The Fabaceae family, also commonly known as Leguminosae, is the third largest plant family after Orchidaceae and Asteraceae, with 730 genera and more than 19,400 species. It includes shrubs, trees and herbaceous plants, which are easily recognized by their fruits (legume) and stipulated leaves. This plant family is mostly found in dry forests and in tropical rainforests [38]. Table 4 presents current knowledge on the extracts of plants from the family Fabaceae and their effect on LOX inhibition.

Souleymane et al. investigated 12-LOX inhibition by dichloromethane and ethyl acetate extracts of *Erythrina senegalensis* roots and stem bark [39]. Ethyl acetate extracts showed higher enzyme inhibition with IC_50_ values of 4.95 and 7.21 μg/mL for the stem bark and roots, respectively. IC_50_ values, concerning dichloromethane extracts, were 21.46 μg/mL for stem bark and 22.03 μg/mL for roots. Root extracts contained more phenolic acids, while stem bark contained more flavonoids, but the inhibition was found to be strongly dependent on the solvent used.

Yoo et al. conducted inhibition of 5-LOX with *Sophora tonkinensis* extract [40]. For the extraction, water ethanol solution (50%, *v*/*v*) was employed. *S. tonkinensis* extract showed strong LOX inhibition (IC_50_ = 1.61 μg/mL). Methanol extract of *Cassia Alata* leaves showed LOX inhibition in the range from 28.3% (at 25.00 μg/mL) to 67.0% (at 100 μg/mL) [41]. Plant polyphenol profile affects the lipoxygenase (LOX)-dependent arachidonic acid metabolism, so the inhibition activity of the extracts could be explained by the polyphenol inhibiting the catalytic activities of LOX [42,43].

Ouédraogo and co-workers extracted the roots of *Pterocarpus erinaceus* in dichloromethane and methanol [44]. Individual components were isolated from dichloromthane (friedelin, 3α-hydroxyfriedelan-2-one, α-sophoradiol and stigmasterol) and methanol (maltol-6-*O*-apiofuranoside-glucopiranoside) extracts. Extracts, as well as individual components, were subjected to LOX inhibition assay. Dichloromethane and methanol extracts inhibited LOX activity for 54.2% and 45.1% at a concentration of 100 μg/mL, respectively. However, individual components were found more active than the extracts themselves. Maltol-6-*O*-apiofuranoside-glucopiranoside, friedelin and α-sophoradio showed a significant LOX inhibition with IC_50_ values of 23.11, 24.44, and 23.91 μg/mL, respectively.

*Crotalaria longipes* plant was extracted in ethanol [45]. LOX activity was checked at different extract concentrations (100–500 mg/mL). The highest inhibition activity (72%) was noticed at 500 mg/mL.

**Table 4:** Lipoxygenase inhibition by extracts of plants of family Fabaceae.

Fabaceae Plants					
Plant Species	Extraction Solvents	Plant Part Extracted	Total Phenolic Content	LOX Inhibition (IC_50_ µg/mL)	References
*Erythrina senegalensis* DC.	Dichloromethane	Stem bark	-	21.46	[39]
	Ethyl acetate			4.95	
	Dichloromethane	Roots	-	22.03	
	Ethyl acetate			7.21	
*Sophora tonkinensis* Gahnep.	Ethanol (50%)	Whole	-	1.61	[40]
*Cassia Alata*	Methanol	Leaves	-	-	[41]
*Pterocarpus erinaceus* Poir.	Dichloromethane	Root barks	-	-	[44]
	Methanol				
*Crotalaria longipes* Wight.	Ethanol	Whole	-	-	[45]

## 6. Lipoxygenase Inhibition by Plant Extracts of Family Lamiaceae

Lamiaceae family is known as a mint family of flowering plants. It contains about 236 genera and 7200 species. The planta are usually aromatic, similar to some culinary herbs (mint, basil, sage, rosemary, oregano, lavender) [46].

Göger et al. investigated 5-LOX inhibition by *Marrubium sivasense* methanolic extract [47]. It was found that extract (at 1 mg/mL) shows poor inhibition of 18.7%. Individual polyphenols components were determined in methanolic extract, namely leucoseptoside A, forsythoside B, verbascoside, martynoside, alyssonoside, as well as glucosides of quercetin, chrysoeriol, and apigenin, and coumaroylglucoside of apigenin.

*Ajuga reptans* and *Ajuga genevensis* are used as traditional medicine for their diuretic, tonic, and astringent activity [48]. Their ethanolic extracts were investigated as potential LOX inhibitors. Obtained results showed that extract of *Ajuga genevensis* is a better 15-LOX inhibitor, but both extracts were found as weak LOX inhibitors with inhibition rates lower than 30.0% at a concentration of 5 mg/mL. *A. genevensis* extract had a higher phenolic and flavonoid content than *A. reptans* extract. Six individual phenolic components (caffeic acid, coumaric acid, rosmarinic acid, luteolin-*O*-glucoside, luteolin, and apigenin) were identified in both extracts. The most abundant compound was luteolin-*O*-glucoside in concentrations of 83.25 and 63.21 µg/g of dry weight for *A. reptans* and *A. genevensis,* respectively.

*Clerodendrum* leaves are used for the treatment of diseases, such as asthma and rheumatism. Phosrithong and Nuchtavorn extracted *Clerodendrum disparifolium* and *Clerodendrum laevifolium* leaves in ethanol and hexane in order to investigate their ability to inhibit 5-LOX activity [49]. Both plant extracts showed great potential in the inhibition of LOX activity. IC_50_ values were more promising for ethanol extract, 24.39 and 14.12 μg/mL, while IC_50_ values in hexane extract were slightly higher, 30.56 and 30.70 μg/mL for *C. disparifolium* and *C. laevifolium*, respectively (Table 5). Total phenolic and flavonoid content were determined in all extracts. Total polyphenols were in the range of 1167.20 to 3344.50 mg GAE/g of dry weight, while flavonoid amount was in the range 9.05–59.91 mg QE/g of dry extract. Ethanol extract with the highest polyphenol content also showed the strongest LOX inhibition.

## 7. Lipoxygenase Inhibition with Plant Extracts of Family Melastomataceae

Plants *Memecylon talbotianum, Memecylon umbellatum,* and *Memecylon malabaricum* belong to the family Melastomataceae. Some of the *Memecylon* species are traditionally used as a cure for skin problems [50].

Leaves of mentioned plants are dried and extracted in methanol in order to examine the 15-LOX inhibition [51]. Obtained IC_50_ values were 29.87, 39.19, and 54.60 μg/mL for *M. malabaricum, M. umbellatum,* and *M. talbotianum*, respectively (Table 6). Estimated amount of phenolic content were 1.12, 1.08, and 1.96 g GAE/100 g dry weight for *M. talbotianum, M. umbellatum,* and *M*. *malabaricum,* respectively. Among the tested extracts, the one with the best LOX inhibitory activity also showed the highest polyphenol content. Afagnigni et al. extracted leaves of *Dissotis multiflora* from the family Melastomataceae in ethanol [52]. Inhibition of 15-LOX was examined at different concentrations of the extract. Obtained result showed that inhibition of 15-LOX was lower than 50% at the concentration of 100 μg/mL.

## 8. Lipoxygenase Inhibition by Plant Extracts of Family Ericaceae

*Gaultheria* plants belong to the Ericaceae family and there is more than 1700 species of this plants. *Gaultheria trichophylla* has blue berries and pink to red flowers. It also known as Himalayan Snowberry. It is commonly used as a folk medicine for the treatment of arthritis. Methanolic and chloroform extracts of *Gaultheria trichophylla* were found to inhibit 5-LOX with 90.5% and 66.9% inhibition at 0.5 mg/mL and IC_50_ values of 277.30 μg/mL and 379.50 μg/mL, respectively (Table 7). *n*-Hexane extract showed lower inhibition of 57.0%. The highest flavonoids (41.30 mg QE equivalent/g) and phenolic (17.50 mg GAE equivalent/g) concentrations were found in methanolic extract [53].

Leaves of *Gaultheria procumbens* L. were extracted in petroleum ether and chloroform [54]. Lipophilic extracts of the wintergreen leaves showed inhibitory effects on LOX with IC_50_ values of 738.50 μg/mL and 899.97 μg/mL in the case of petroleum ether and chloroform extract, respectively. The performed GC-MS analysis of petroleum ether extracts revealed the presence of four major compound groups (aliphatic hydrocarbons, alcohols, carboxylic acids, and terpenoids). The docanose and octacosane were present in the highest amounts. Simple phenols and terpenoids were also present in significant percentage (methyl benzoate, ursolic acid, α-amirin, *β*-sitosterol, methyl salicylate, and oleanolic acid). Chloroform extract contained a lower amount of alcohol, simple aliphatic hydrocarbons, and carboxylic acid compared with petroleum ether extract. Dominant compounds in chloroform extract were ursolic acid, methyl benzoate, methyl salicylate, and oleanolic acid.

It could be noticed that all examined extracts from the family Ericaceae had poor LOX inhibition with high IC_50_ values.

## 9. Lipoxygenase Inhibition by Plant Extracts of Family Menispermaceae

Plant *Cyclea Barbarata* belong to the plant family of Menispermaceae. Leaves of this plant contain many secondary metabolites, such as flavonoids, steroids, tannins, and saponins. It was reported that flavonoids have many benefits, such as antibacterial, anti-inflammatory, anticancer, antioxidant, hepatoprotective, and antiviral properties [55,56]. Handayani et al. reported inhibition of LOX with leaf extracts of *C. Barbarata* [57]. Three various solvents (ethyl acetate, *n*-hexane, and methanol) were employed for extraction. Among the investigated extracts, only ethyl acetate extract showed significant inhibition of LOX with an IC_50_ value of 0.26 µg/mL (Table 8). Total flavonoids content of ethyl acetate extracts was 21.62 mg QE/g.

LOX inhibition activity of ethanolic extracts of *Sphenocentrum jollyanum* was investigated by Sinbad et al. [58]. *S. jollyanum* possesses many biological and pharmaceutical properties, including antimalarial, antioxidant, anti-inflammatory, antidiabetic, and anti-angiogenic. Roots and stems were extracted and both extracts showed poor inhibition of LOX enzyme with IC_50_ values of 541.00 and 426.00 µg/mL, respectively.

## 10. Lipoxygenase Inhibition by Plant Extracts of Family Rosacea

*Cydonia oblonga* is a plant from the Rosacea family and it is a rich source of antioxidants. Phytochemical screening revealed the presence of cardiac glycosides, alkaloids, terpenoids, tannins, flavonoids, saponins, and steroids [59]. Berkoz determined lipoxygenase inhibition and total phenolic content in the crude extract, aqueous and organic fractions (chloroform, ethyl acetate, butanol) of dry fruit of *Cydonia oblonga* [60]. The best LOX inhibition was achieved by methanol extract (IC_50_ = 99.30 µg/mL), which showed the highest phenolic content of 367.00 mg GAE/g of extract. Other extracts also appeared to be the prominent inhibitors of LOX with IC_50_ values in the range of 101.80 to 227.30 µg/mL (Table 9).

Similar research was conducted by Marchelak and co-workers [61]. *Prunus Spinosa* flowers are known as traditional medicine for treatment of inflammation, urinary tract disorders, and cardiovascular diseases. Flowers of *P. spinosa* were subjected to the extraction in five different solvents. Up to 57 constituents, mostly phenolic acids, flavonoids, and proantocyanidins were identified in these extracts. Ethyl acetate extract showed the strongest inhibition of LOX with IC_50_ value of 135.36 µg/mL and had high polyphenol content of 584.07 mg GAE/g of dry weight. Total flavonoid amounts of extracts were in the range of 1.88 to 490.63 mg/g of dry weight.

## 11. Lipoxygenase Inhibition by Plant Extracts of Family Sapindaceae

*Paullinia pinnata* is a plant from family Sapindaceae, whose phytochemical profile showed the presence of numerous secondary metabolites [52]. Different plant parts showed various activities, such as antioxidant, antibacterial, antidiarrheal, anti-inflammatory, antityphoid, and anxiolytic proprties. Ethanolic extracts (50% ethanol) of *P. pinnata* showed poor 15-lipoxygenase inhibitory potential.

Ferreres et al. extracted leaves and stem bark of *Allophylus africanus* with water [62]. Both extracts showed good LOX inhibitory activity with IC_50_ values of 41.28 and 107.77 µg/mL for leaves and stem bark, respectively (Table 10). In aqueous leaf extracts, 30 flavones were identified, with the dominant ones being apigenin derivatives and luteolin derivatives. Stem bark was characterized by mono-*C*-glycosides-*O*-glycosylated and apigenin di-*C*-glycosides.

## 12. Lipoxygenase Inhibition by Plant Extracts of Other Plant Families

Many authors reported inhibition of lipoxygenase enzymes by plant extracts from various plant families, as listed in Table 11. Plant extracts varied from poor to excellent LOX inhibitors.

Hexan seed extract of *Cannabis sativa* L. (Cannabaceae) showed poor LOX inhibition, lower than 50.0% [63]. Salleh et al. reported inhibition of LOX enzyme by *Beilschmiedia penangiana* (Lauraceae) leaves and stem bark extracts [64]. Plant parts were extracted in hexane, ethyl acetate, and methanol. Leaf extracts inhibited lipoxygenase for 39.8%, 45.8%, and 32.4% in hexane, ethyl acetate, and methanol, respectively. Stem bark also showed moderate inhibition of 24.2%, 41.5%, and 46.3%, respectively. Shafiri-Rad et al. reported LOX inhibition of 55.3% (methanolic extract of *Veronica persica* (Plantaginaceae) at concentration of 1 mg/mL [65]. Leaf extracts of *Kigelia Africana* (Bignoniaceae) showed a lipoxygenase inhibition at a concentration of 500 µg/mL [66]. Ethanolic, aqueous, butanol, ethyl acetate, and hexane fractions showed LOX inhibition of 91.7%, 85.8%, 84.8%, 91.6%, and 86.1%, respectively. Furthermore, methanol seed extract of *Nigella sativa* (Ranunculaceae) and ethanol extract of *Petiveria alliacea* (Phytolaccaceae) showed LOX inhibition of 58.4% and 65.0%, at a concentration of 500 µg/mL, respectively [67,68]. *Archidium ohioense* (Archidiaceae) plant was extracted in four organic solvents and water [69]. Ethyl acetate and dichloromethane extract were the most active and showed inhibition against LOX of 75.0% and 71.6% at a concentration of 50 µg/mL, respectively.

Eshwarppa et al. employed four solvents (petroleum ether, chloroform, ethanol, and water) in the extraction of *Terminalia chebula* (Combretaceae) leaves [70]. Minimum inhibitory concentrations (MIC) were 560.0, 673.0, 726.0, and 847.0 µg/mL for ethanol, water, chloroform, and petroleum ether, respectively. Total phenolic content was in the range of 51.00 to 141.00 mg GAE/g of dry weight and total flavonoids in the range of 39.00 to 125.00 mg QE/g of dry weight.

Extraction of leaves and stem bark of *Ficus curtipes* (Moraceae) was carried out in methanol [71]. Stem extract showed strong inhibition against LOX with an IC_50_ value of 10.75 µg/mL, while leaf extract had significantly poorer IC_50_ value (IC_50_ = 200.80 µg/mL). Huh et al. extracted *Fraxinus rhynchophylla* (Oleaceae) leaves, outer bark, and endodermis in ethanol [72]. Only outer bark had LOX inhibitory activity with an IC_50_ value of 62.60 µg/mL. Furthermore, Alam et al. carried out extractions of *Zanthoxylum armatum* (Rutaceae) leaves, bark, and fruit in methanol [73]. Bark and fruit extracts showed very good inhibition of LOX, with IC_50_ values of 90.50 and 70.30 µg/mL. Fruit extracts had the higher phenolic amount (26.50 mg GAE/g of extract) than bark extracts (15.50 mg GAE/g of extract). Leaves and roots of *Bruguiera cylindrica* (L.) (Rhizophoraceae) were extracted in two organic solvents (methanol and dichloromethane) [74]. All extract showed LOX inhibition, except for the methanolic extracts of leaves. The best inhibition was accomplished in dichloromethane leaf extract (IC_50_ = 45.70 µg/mL), followed by dichloromethane root extracts (IC_50_ = 64.00 µg/mL) and methanol root extracts (IC_50_ = 71.0 µg/mL). Endosperm extracts of *Arenga pinnata* (Arecaceae) showed LOX inhibition of 71.37 µg/mL (IC_50_) [75].

Many authors reported extraction of plant materials in different organic solvents (ethanol, methanol, chloroform, dichloromethane, hexane, cyclohexane, ethyl acetate, butyl methyl ether) and water, which are tested on LOX inhibitory potential [76,77,78,79,80,81]. Langhansova et al. employed four solvents in the extraction of *Myrica rubra* (Myricaceae) leaves [76]. The best inhibition of lipoxygenase was achieved with the ethyl acetate extract (IC_50_ = 8.30 µg/mL). Extracts of a *Averrhoa carambola* L. (Oxalidaceae) and *Polygonatum verticillatum* (Asparagaceae) also showed the strongest activities against LOX in ethyl acetate with IC_50_ values of 7.84 and 97.0 µg/mL, respectively [77,79]. On the other hand, methanolic extracts of *Piper stylosum* (Piperaceae) and *Boswellia dalzielii* (Burseraceae) showed the best LOX inhibition with IC_50_ values of 80.50 and 28.10 µg/mL, respectively [78,80]. Dichloromethane was proven the best extraction solvent in the case of *Jatropha gossypifolia* (Euphorbiaceae) [81]. With dichloromethane extracts, IC_50_ value of 57.10 µg/mL was obtained for LOX inhibition.

LOX inhibition of water extract of *Lavatera cretica* L. (Malvaceae) should also be mentioned [82]. Leaf and flower extracts showed very strong inhibition of LOX, with IC_50_ values of 0.01 µg/mL. Extracts had high polyphenolic content of 254.62 and 191.38 mg GAE/g dry weight.

## 13. Conclusions

This paper gives an overview of the effect of various plant extracts of various plant families on LOX inhibition. It turned out that soybean lipoxygenases, both 5-LOX and 15-LOX (found in mammalians), were successfully inhibited by plant extracts. Ethanol extract of *Cnicus benedictus* showed great inhibition of 15-LOX with an IC_50_ value of 52.7 µg/mL. The best inhibitor of 5-LOX was the ethanolic extract of *Sophora tokinensis* with an IC_50_ value of 1.61 µg/mL. Soybean lipoxygenase was successfully inhibited by *Lavatera cretica* leaf and flower water extracts with an IC50 value of 0.01 µg/mL.

Based on the abovementioned, it becomes clear that the selection of appropriate solvents for phytochemical extractions, as well as the corresponding phytochemical profiles, play important roles in LOX inhibition. While some of the extracts show excellent inhibitory activities, others seem poorly effective in LOX inhibition. Moreover, several reports shows greater LOX inhibitory activity of isolated compounds themselves, than extracts on the whole. Therefore, it seems that there is lot of space to fill in the gaps in regards to finding the most novel, prominent LOX inhibitors among the multitude of plant phytochemicals.

## Figures and Tables

**Table 1:** Lipoxygenase inhibition by extracts of plants of family Apiaceae.

Apiaceae Plants					
Plant Species	Extraction Solvents	Plant Part Extracted	Total Phenolic Content	LOX Inhibition (IC_50_ µg/mL)	References
*Alepidea amatymbica* Eckl. & Zeyh	Acetone	Roots	-	-	[16]
*Apium graveolens* Linn.	Methanol (60%)	Seeds	0.34 ^1^	-	[17]
*Petroselinum crispum* Mill.			0.29 ^1^	-	
*Bupleurum marginatum* Wall.	Methanol	Aerial parts	-	45.28	[18]
	Dichloromethane			25.92	
*Scandix pecten-veneris* L.	Methanol (85%, *v*/*v*)	Leaves	377.94 ^2^	641.57	[19]
*Pituranthos chloranthus* Benth.	Water	Aerial parts	1.36 ^2^	20.00	[20]
*Eryngium planum* L.	Ethanol (50%, *v*/*v*)	Aerial parts	1.88 ^1^	31.30	[21]
*Levisticum officinale* W.D.J. Koch	Ethanol:water:HCl (70:29:1 *v*/*v*/*v*)	Leaves	-	-	[22]
*Anethum graveolens* L.	Water	Aerial parts	67.22 ^1^	43.00	[23]
		Roots			
*Foeniculum vulgare* Mill.	Water	Aerial parts	35.76 ^1^	52.00	
		Roots			
*Pimpinella anisum* L.	Water	Aerial parts	29.08 ^1^	35.00	
		Roots			

^1^ results are expressed as mg gallic acid equivalent (GAE)/mL of extract. ^2^ results are expressed as mg gallic acid equivalent (GAE)/g of extract.

**Table 2:** Lipoxygenase inhibition by extracts of plants of the family Asteraceae.

Asteraceae Plants					
Plant Species	Extraction Solvents	Plant Part Extracted	Total Phenolic Content	LOX Inhibition (IC_50_ µg/mL)	References
*Matricaria chamomilla* L.	Methanol (60%)	Flowers	458.10 ^1^	-	[17]
*Artemisia vulgaris* L.	Water	Aerial parts	0.91 ^2^	40.00	[20]
*Cnicus benedictus* L.	Ethanol (50%)	Aerial parts	2.65 ^1^	52.7	[21]
*Artemisia nilagirica* C.B. Clarke	*n*-Hexane Methanol Ethanol	Leaves	-	128.20	[27]
*Chrysanthemum indicum* L.	Chloroform	Whole	1.65 ^3^	-	[28]
	Dichloromethane		3.14 ^3^		
	*n*-Hexane		0.90 ^3^		
	Methanol		11.85 ^3^		
*Tagetes erecta* L.	Chloroform	Whole	6.97 ^3^	-	
	Dichloromethane		4.18 ^3^		
	*n*-Hexane		5.37 ^3^		
	Methanol		15.67 ^3^		
*Vernonia oligocephala* DC.	Methanol (80%)	Roots	113.11 ^2^	>500	[29]
	Dichloromethane		59.08 ^2^	>500	
	Ethyl acetate		27.01 ^2^	>500	
	*n*-Hexane		4.76 ^2^	>500	
	*n*-Butanol		3.12 ^2^	132.21	
*Tanacetum cilicicum* Boiss.	Ethanol	Capitula	87.01 ^2^	156.00	[30]
	*n*-Hexane		10.28 ^2^	167.50	
	Chloroform		72.37 ^2^	127.00	
	Ethyl acetate		174.10 ^2^	9.44	
	Ethanol (60%, *v*/*v*)		42.45 ^2^	240.90	
	Ethanol	Stems	28.44 ^2^	255.70	
	*n*-Hexane		6.11 ^2^	202.90	
	Chloroform		68.85 ^2^	112.40	
	Ethyl acetate		77.36 ^2^	395.10	
	Ethanol (60%, *v*/*v*)		12.08^2^	360.30	
	Ethanol	Leaves	57.58 ^2^	187.40	
	*n*-Hexane		13.63 ^2^	149.80	
	Chloroform		63.28 ^2^	148.10	
	Ethyl acetate		175.60 ^2^	85.18	
	Ethanol (60%, *v*/*v*)		40.59 ^2^	220.30	
*Galatella tatarica* Less.	Methanol	Whole	0.41 ^1^	-	[31]
*Galatella villosa* L.	Methanol	Whole	0.39 ^1^	-	
*Cota fulvida* Holub.	Methanol	Aerial parts	0.29 ^1^	-	[32]

^1^ results are expressed as mg gallic acid equivalent (GAE)/mL extract; ^2^ results are expressed as mg gallic acid equivalent (GAE)/g extract; ^3^ results are expressed as g gallic acid equivalent (GAE)/100 g dry extract.

**Table 3:** Lipoxygenase inhibition by extracts of plants of family Clusiaceae.

Clusiaceae Plants					
Plant Species	Extraction Solvents	Plant Part Extracted	Total Phenolic Content	LOX Inhibition (IC_50_ µg/mL)	References
*Garcinia porrecta* Laness.	Methanol	Stem bark	-	0.23	[34]
	Ethyl acetate			0.52	
	*n*-Hexane			4.87	
*Garcinia hombroniana* Pierre	Methanol	Leaves	-	1.31	[35]
	Ethyl acetate			0.13	
	*n*-Hexane			2.05	
*Garcinia lateriflora* Blume	Methanol	Leaves	-	0.69	[36]
	Ethyl acetate			0.79	
	*n*-Hexane			1.31	
*Garcinia kydia* Roxburgh	Methanol	Leaves	-	0.5	[37]
	Ethyl acetate			0.21	
	*n*-Hexane			3.57	

**Table 5:** Lipoxygenase inhibition by extracts of plants of family Lamiaceae.

Lamiaceae Plants					
Plant Species	Extraction Solvents	Plant Part Extracted	Total Phenolic Content	LOX Inhibition (IC_50_ µg/mL)	References
*Marrubium sivasense* Aytaç, Akgül & Ekici	Methanol	Aerial parts	-	-	[47]
*Ajuga genevensis* L.	Ethanol (70%)	Whole	354.70 ^2^	-	[48]
*Ajuga reptans* L.			296.50 ^2^	-	
*Clerodendrum disparifolium* Blume	Ethanol	Leaves	1167.20 ^1^	24.39	[49]
	*n*-Hexane		1272.40 ^1^	30.56	
*Clerodendrum laevifolium* Blume	Ethanol	Leaves	3344.50 ^1^	14.12	
	*n*-Hexane		1611.00 ^1^	30.70	

^1^ results are expressed as mg gallic acid equivalent (GAE)/g dry extract; ^2^ results are expressed in mg gallic acid %.

**Table 6:** Lipoxygenase inhibition by extracts of plants of family Melastomataceae.

Melastomataceae Plants					
Plant Species	Extraction Solvents	Plant Part Extracted	Total Phenolic Content	LOX Inhibition (IC_50_ µg/mL)	References
*Memecylon talbotianum* Brandis	Methanol	Leaves	1.12 ^1^	34.60	[51]
*Memecylon malabaricum* Cogn.	Methanol	Leaves	1.96 ^1^	29.87	
*Memecylon umbellatum* Burm. f.	Methanol	Leaves	1.08 ^1^	39.19	
*Dissotis multiflora* Sm.	Ethanol	Leaves	-	-	[52]

^1^ results are expressed as g gallic acid equivalent (GAE)/100 g dry weight.

**Table 7:** Lipoxygenase inhibition by extracts of plants of family Ericaceae.

Ericaceae Plants					
Plant Species	Extraction Solvents	Plant Part Extracted	Total Phenolic Content	LOX Inhibition (IC_50_ µg/mL)	References
*Gaultheria trichophylla* Royle	Methanol	Whole	17.50 ^1^	277.30	[53]
	Chloroform		5.00 ^1^	379.50	
	*n*-Hexane		3.20 ^1^	448.00	
*Gaultheria procumbens* L.	Petroleum ether	Leaves	-	738.49	[54]
	Chloroform			899.97	

^1^ results are expressed as mg gallic acid equivalent (GAE)/g of extract.

**Table 8:** Lipoxygenase inhibition by extracts of plants of family Menispermaceae.

Menispermaceae Plants					
Plant Species	Extraction Solvents	Plant Part Extracted	Total Phenolic Content	LOX Inhibition (IC_50_ µg/mL)	References
*Cyclea Barbata* Miers.	Ethyl acetate	Leaves	-	0.26	[57]
	*n*-Hexane			-	
	Methanol			-	
*Sphenocentrum jollyanum* Pierre.	Ethanol (70%)	Roots	-	541.00	[58]
		Stem		426.00	

**Table 9:** Lipoxygenase inhibition by extracts of plants of family Rosacea.

Rosacea Plants					
Plant Species	Extraction Solvents	Plant Part Extracted	Total Phenolic Content	LOX Inhibition (IC_50_ µg/mL)	References
*Cydonia oblonga* Mill.	Methanol (70%)	Fruit	367.00 ^1^	99.30	[60]
	Water		115.30 ^1^	101.80	
	Ethyl acetate		65.00 ^1^	227.30	
	Chloroform		244.90 ^1^	102.40	
	Butanol		123.40 ^1^	207.00	
*Prunus spinosa* L.	Methanol (70%)	Flower	206.07 ^2^	327.36	[61]
	Diethyl ether		464.57 ^2^	150.36	
	Ethyl acetate		584.07 ^2^	135.36	
	*n*-Butanol		296.57 ^2^	171.10	
	Water		64.60 ^2^	479.50	

^1^ results are expressed as mg gallic acid equivalent (GAE)/g of extract. ^2^ results are expressed as mg gallic acid equivalent (GAE)/g of dry weight.

**Table 10:** Lipoxygenase inhibition by extracts of plants of family Sapindaceae.

Sapindaceae Plants					
Plant Species	Extraction Solvents	Plant Part Extracted	Total Phenolic Content	LOX Inhibition (IC_50_ µg/mL)	References
*Paullinia pinnata* L.	Ethanol (95%)	Leaves	-	-	[52]
*Allophylus africanus* P. Beauv.	Water	Leaves	-	41.28	[62]
		Stem bark		107.77	

**Table 11:** Lipoxygenase inhibition by extracts of different plant families and species.

Plant Family/Species	Extraction Solvents	Plant Part Extracted	Total Phenolic Content	LOX Inhibition (IC_50_ µg/mL)	References
Cannabaceae/*Cannabis sativa* L.	*n*-Hexane	Seeds	-	-	[63]
Lauraceae/*Beilschmiedia penangiana* Gamble	*n*-Hexane	Leaves	101.50 ^3^	-	[64]
	Ethyl acetate		66.80 ^3^		
	Methanol		59.60 ^3^		
	*n*-Hexane	Stem bark	47.70 ^3^	-	
	Ethyl acetate		46.30 ^3^		
	Methanol		176.80 ^3^		
Plantaginaceae/*Veronica persica* Poir.	Methanol (70%)		-	-	[65]
Bignoniaceae/*Kigelia Africana* (Lam.) Benth.	Ethanol (70%)	Leaves	7.84 ^3^	-	[66]
	*n*-Hexane		5.73 ^3^		
	Ethyl acetate		22.55 ^3^		
	*n*-Butanol		18.77 ^3^		
	Water		12.67 ^3^		
Ranunculaceae/*Nigella sativa* L.	Methanol	Seeds	-	-	[67]
Phytolaccaceae/*Petiveria alliacea* L.	Ethanol	Whole	-	-	[68]
Archidiaceae/*Archidium ohioense* Schimp. Ex Müll. Hal.	Dichloromethane	Whole	-	-	[69]
	Ethyl acetate		-	-	
Combretaceae/*Terminalia chebula Retz.*	Petroleum ether	Leaves	107.00 ^4^	-	[70]
	Chloroform		51.00 ^4^		
	Water		95.00 ^4^		
	Ethanol		141.00 ^4^		
Moraceae/*Ficus curtipes* Corner.	Methanol	Leaves		200.80	[71]
		Stem bark		10.75	
Oleaceae *Fraxinus rhynchophylla* Roxb.	Ethanol	Leaves	-	-	[72]
		Outer bark		62.60	
		Endodermis		-	
Rutaceae/*Zanthoxylum armatum* DC.	Methanol	Leaves	13.10 ^3^	-	[73]
		Bark	15.50 ^3^	90.50	
		Fruit	25.60 ^3^	70.30	
Rhizophoraceae/*Bruguiera cylindrica* L.	Methanol	Leaves	-	-	[74]
	Dichloromethane			45.70	
	Methanol	Roots		71.00	
	Dichloromethane			64.00	
Arecaceae/*Arenga Pinnata* (Wurmb.) Merr.	Ethanol (95%)	Endosperm	-	71.37	[75]
Myricaceae/*Myrica rubra* Siebold & Zucc.	Water	Leaves	209.83	50.57	[76]
	*n*-Hexane		18.41	-	
	Ethyl acetate		192.39	8.30	
	*tert*-Butylmethylether		379.50	32.32	
Oxalidaceae/*Averrhoa carambola* L.	70% Ethanol	Leaves	-	37.00 ^1^	[77]
	Ethyl acetate			7.84 ^1^	
	*n*-Hexane			107.71 ^1^	
	Water			64.05 ^1^	
Piperaceae/*Piper stylosum* Miq.	Methanol	Aerial parts	-	80.50 ^2^	[78]
	Ethyl acetate			82.80 ^2^	
	*n*-Hexane			85.20 ^2^	
Asparagaceae/*Polygonatum verticillatum* All.	Methanol	Aerial parts		125.00	[79]
	*n*-Hexane			-	
	Chloroform		-	122.00	
	Ethyl acetate			97.00	
	*n*-Butanol			116.00	
	Water			109.00	
Burseraceae/*Boswellia dalzielii* Hutch	Dichloromethane	Leaves	48.23 ^4^	58.01	[80]
	Ethyl acetate		148.81 ^4^	45.10	
	Methanol		315.97 ^4^	28.01	
	Cyclohexane		3.58 ^4^	-	
Euphorbiaceae/*Jatropha gossypifolia* L.	Methanol	Leaves		162.50	[81]
	*n*-Hexane			-	
	Dichloromethane			57.10	
	Ethyl acetate			58.50	
	Butanol			59.40	
Malvaceae/*Lavatera cretica* L.	Water	Leaves	254.62 ^4^	0.01	[82]
		Flowers	191.38 ^4^	0.01	
		Bract + sepal	189.60 ^4^	0.15	
		Seeds	43.69 ^4^	-	

^1^ results are expressed in ppm; ^2^ results expressed in µM; ^3^ results are expressed as mg gallic acid equivalent (GAE)/g of extract; ^4^ results are expressed as mg gallic acid equivalent (GAE)/g of dry mass.

## Data Availability

No new data were created or analyzed in this study. Data sharing is not applicable to this article.
